# Glycemic Status, Stent Count, and In-Stent Restenosis: Exploratory Decomposition and Sex-Related Analyses in a Retrospective Cohort

**DOI:** 10.3390/jcm15218685

**Published:** 2026-11-07

**Authors:** Yuan Wang, Yan Zhuang, Liao Zhang, Haiyan Song

**Affiliations:** Department of Endocrinology and Metabolism, The Second Affiliated Hospital of Harbin Medical University, Harbin 150001, China; 2024021302@hrbmu.edu.cn (Y.W.); zy_03212023@163.com (Y.Z.); zhangl990129@163.com (L.Z.)

**Keywords:** fasting blood glucose, in-stent restenosis, stent count, stent underexpansion, sex-specific analysis

## Abstract

**Background/Objectives:** Fasting blood glucose (FBG) and stent count have been associated with in-stent restenosis (ISR), but their interrelationships remain unclear. We evaluated the FBG–ISR association and explored baseline stent count as a potential mediator. **Methods:** This single-center retrospective cohort comprised 510 patients with follow-up angiography after percutaneous coronary intervention (PCI). Analyses included multivariable regression, exploratory decomposition in 468 patients sampled before PCI, and intravascular imaging analyses in 284 patients. **Results:** ISR was observed in 56 patients (11.0%). The adjusted FBG–ISR odds ratio (OR) was 1.100 per mmol/L (95% confidence interval [CI] 1.001–1.208; *p* = 0.048); Firth and diabetes-adjusted CIs included 1. For FBG increasing from 5.07 to 7.59 mmol/L, the indirect component associated with baseline stent count was 0.48 percentage points (95% CI 0.06 to 1.05); the direct and total CIs included zero. Exploratory analyses suggested higher direct-effect estimates in men and positive indirect-effect estimates in women; between-sex indirect differences remained uncertain. Higher stent count was associated with underexpansion, which was associated with ISR (adjusted OR 4.47, 95% CI 1.85–10.78). **Conclusions:** Higher FBG showed a modest positive association with ISR, with uncertainty across sensitivity analyses. Exploratory analyses yielded a positive indirect estimate for stent count and linked underexpansion to ISR.

## 1. Introduction

Diabetes mellitus affected an estimated 589 million adults aged 20–79 years worldwide in 2024, corresponding to a prevalence of 11.1%, with the number projected to reach 853 million by 2050 [1].

Although drug-eluting stents have substantially reduced restenosis after percutaneous coronary intervention (PCI), in-stent restenosis (ISR) remains an important cause of stent failure and repeat revascularization [2,3,4,5]. Both biological and mechanical factors contribute to ISR. Admission or periprocedural hyperglycemia, higher fasting blood glucose (FBG), and poor long-term glycemic control have been linked to ISR or coronary stent failure [6,7,8,9], as have lesion and procedural characteristics such as a greater number of implanted stents, longer stented segments, smaller vessel caliber, and stent underexpansion [2,10,11,12,13,14,15].

However, the interrelationships among FBG, baseline stent count, and ISR remain unclear. Diabetes is associated with more diffuse coronary disease [16], while baseline stent count partly reflects both disease extent and PCI treatment. Because women and men differ in coronary vessel caliber, plaque characteristics, vascular remodeling, and PCI treatment [17,18,19,20,21], we also explored sex-specific estimates.

We therefore evaluated the association between FBG and ISR in patients who underwent PCI and follow-up coronary angiography. Using exploratory statistical decomposition, we estimated a component associated with baseline stent count and a remaining component, and explored sex-specific estimates. We also examined patient-level associations among baseline stent count, stent underexpansion, and ISR using optical coherence tomography (OCT) and/or intravascular ultrasound (IVUS) records.

## 2. Materials and Methods

### 2.1. Study Design and Population

**Figure 1:**
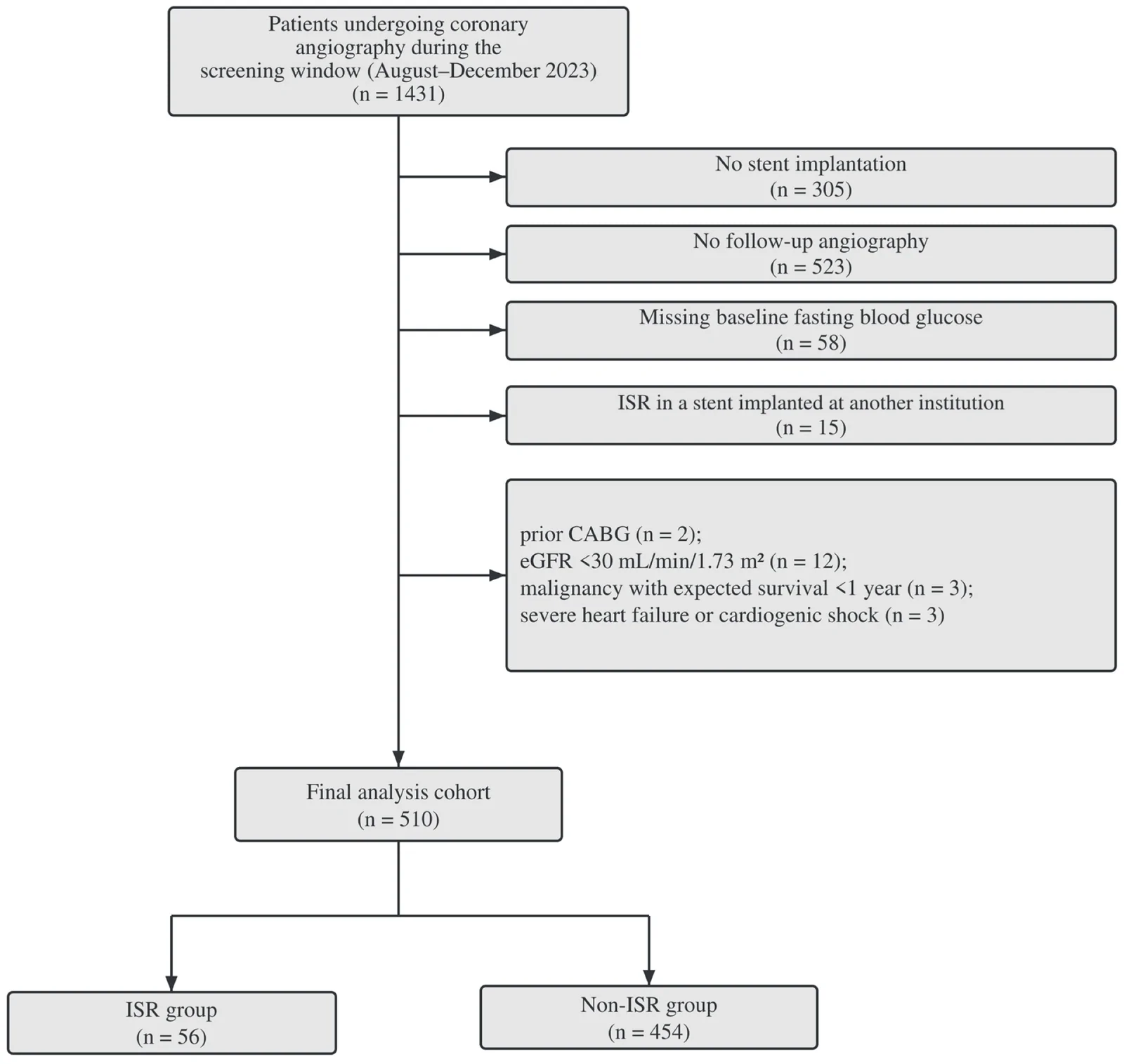
Patient selection flowchart. Of 1431 screened patients, 921 were excluded, and 510 were analyzed (454 without ISR and 56 with ISR). CABG, coronary artery bypass grafting; eGFR, estimated glomerular filtration rate; ISR, in-stent restenosis.

This single-center retrospective cohort study used a fixed screening window to identify the source population. We screened 1431 patients who underwent coronary angiography in two cardiology wards at The Second Affiliated Hospital of Harbin Medical University between August and December 2023 (Figure 1).

**Figure 2:**
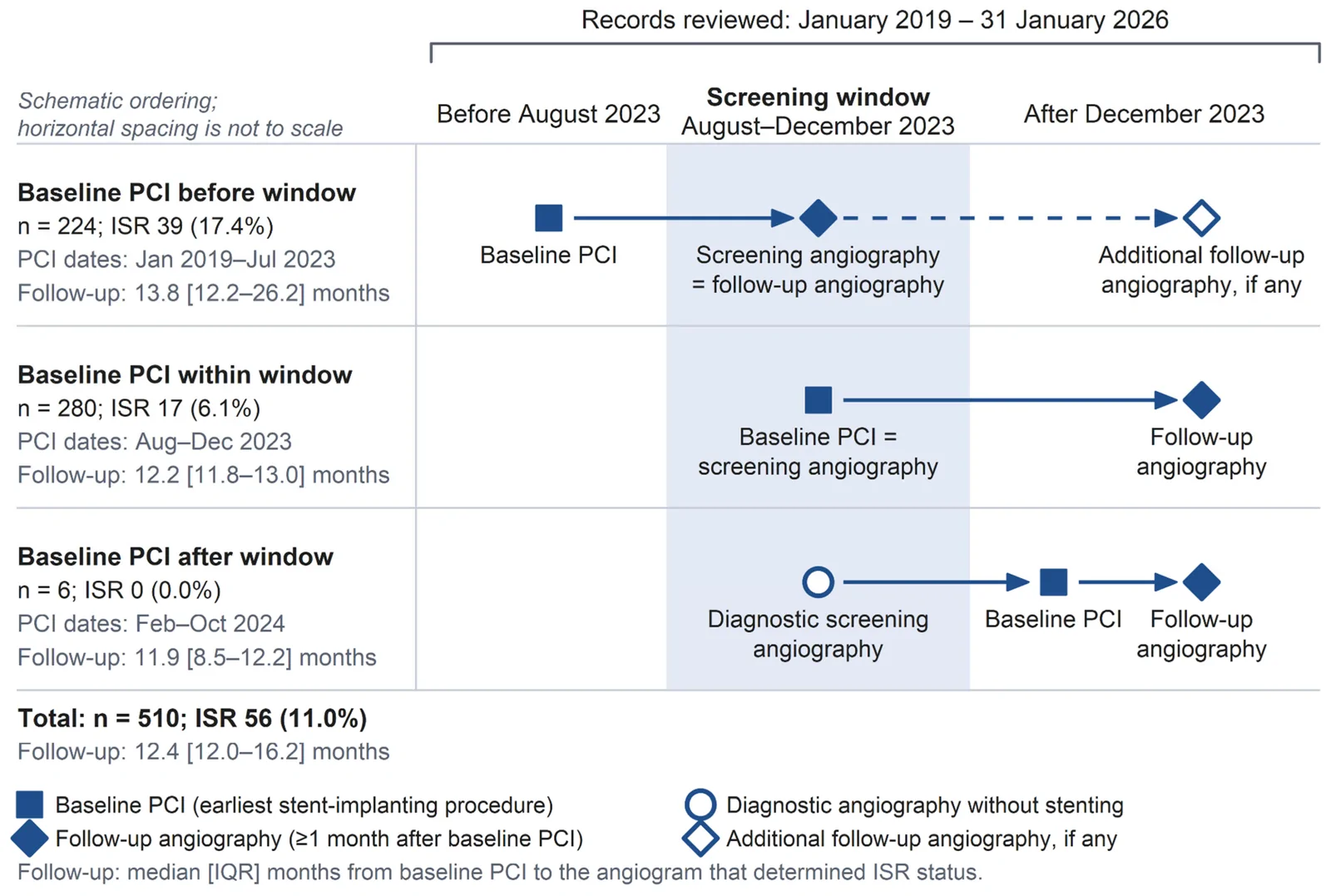
Timing of baseline PCI and follow-up angiography relative to the 2023 screening window. Patients are grouped by the timing of baseline PCI relative to the screening window. Patient numbers, ISR events, and follow-up duration (median [IQR] months) are shown for each group. Follow-up extends to the first angiogram showing ISR or the last angiogram in patients without ISR. Horizontal spacing is not to scale.Solid arrows indicate the main temporal sequence of angiography and PCI events, whereas the dashed arrow indicates possible additional follow-up angiography, if any.IQR, interquartile range; PCI, percutaneous coronary intervention; ISR, in-stent restenosis.

For these patients, all angiography and PCI records in the hospital information system from January 2019 through 31 January 2026 were reviewed to identify those with stent implantation and at least one eligible follow-up angiogram. Baseline PCI was defined as the earliest procedure with stent implantation in the reviewed records and could occur before, during, or after the screening window (Figure 2). Procedures within 1 month of baseline PCI, including staged procedures, formed the baseline treatment episode; angiography performed more than 1 month later was considered follow-up.

Exclusion criteria were no stent implantation, no follow-up angiography, missing baseline FBG, ISR attributable to a stent implanted at another institution, previous coronary artery bypass grafting, an estimated glomerular filtration rate < 30 mL/min/1.73 m^2^, malignancy with an expected survival < 1 year, or severe heart failure or cardiogenic shock. The final cohort comprised 510 patients (Figure 1). The Ethics Committee of The Second Affiliated Hospital of Harbin Medical University approved the study (YJSKY2025-143) and waived the requirement for informed consent due to the retrospective design and use of de-identified clinical records.

### 2.2. Exposure, Outcome, and Covariates

Baseline data were obtained from records of the hospitalization during which baseline PCI was performed. FBG was measured in venous blood collected on the morning after admission following an overnight fast. Based on the available records, baseline PCI preceded blood sampling in 42 patients; the remaining 468 patients formed the pre-PCI sampling subset. In these 468 patients, the median interval from blood sampling to baseline PCI was 28.2 h (IQR 10.0–62.3). Diabetes was defined as a previous diagnosis or a baseline glycated hemoglobin (HbA1c) level ≥ 6.5%; diabetes subtype could not be reliably classified from the available records. Biological sex was obtained from medical records. Body mass index (BMI) was calculated as weight (kg) divided by height (m) squared using measurements recorded at baseline. Total stent count was the number of stents implanted during the baseline treatment episode; stents implanted afterward, including those placed to treat ISR, were not counted. All stents implanted during the baseline treatment episode were second-generation drug-eluting stents. Diseased segment count was defined as the number of coronary segments with >50% stenosis on the baseline angiogram.

ISR was defined as ≥50% diameter stenosis within the stent or within 5 mm of either stent edge on follow-up angiography [2]. The outcome was ISR in at least one stent implanted during the baseline treatment episode. Two observers independently assessed the angiograms, with disagreements adjudicated by a third observer. Follow-up continued through 31 January 2026. Follow-up duration was calculated from baseline PCI to the first angiogram showing ISR in patients with ISR, or to the last follow-up angiogram in patients without ISR.

### 2.3. Intravascular Imaging Analysis

Among the 510 patients, 284 (203 men and 81 women) had evaluable OCT and/or IVUS records, including 28 with ISR; 225 had OCT records. Two physicians independently reviewed the imaging records, and any disagreements were adjudicated by a third physician. Stent underexpansion, malapposition, stent-edge dissection or tear, tissue prolapse, plaque rupture, and thrombus were classified according to explicit descriptions in the original reports. Each finding was coded as present or absent at the patient level.

### 2.4. Statistical Analysis

Continuous variables are presented as medians [interquartile ranges] and categorical variables as counts (percentages). Groups were compared using the Mann–Whitney U test, chi-square test, or Fisher’s exact test, as appropriate. The association between FBG (per 1 mmol/L) and ISR was evaluated using logistic regression. Model 1 was unadjusted. Model 2 adjusted for age, sex, current smoking, and follow-up duration. Model 3 additionally adjusted for left ventricular ejection fraction (LVEF) and ST-segment elevation myocardial infarction (STEMI). In restricted cubic spline models, knots were placed at the 10th, 50th, and 90th percentiles of FBG (4.62, 5.79, and 10.37 mmol/L), with 5.79 mmol/L as the reference. These models used the same covariates as Model 3. Associations were also estimated within age (<65 and ≥65 years), sex, and diabetes subgroups. We tested interactions between FBG and age group, sex, or diabetes status in Model 3.

Firth’s penalized-likelihood logistic regression was used as a sensitivity analysis of Model 3 with the same covariates. Odds ratios, 95% confidence intervals, and two-sided *p* values were reported.

Missing LVEF values (112 patients, 22.0%) and BMI values (2 patients) were multiply imputed using chained equations with predictive mean matching (10 datasets). Imputation was applied to all 510 patients, and the 468 patients sampled before PCI were then selected for decomposition analyses. The imputation models included ISR, FBG, total stent count, and the analysis covariates. Regression estimates were pooled using Rubin’s rules. Other variables used in the principal analyses had no missing values.

We performed an exploratory statistical decomposition within a counterfactual mediation framework in the pre-PCI sampling subset (*n* = 468). Component associations were estimated using Poisson regression with HC0-robust standard errors for FBG–stent count and logistic regression for ISR. The mediator model included FBG, age, sex, current smoking, LVEF, STEMI, and diseased segment count. The outcome model included FBG, total stent count, the same covariates, and follow-up duration.

Using g-computation, we decomposed the FBG–ISR association into an indirect effect associated with stent count (ACME) and a remaining direct effect (ADE) [22,23,24]. Their sum was the total effect (TE). Estimates were reported as risk differences in percentage points for an increase in FBG from 5.07 to 7.59 mmol/L, corresponding to the 25th and 75th percentiles in the full cohort.

Point estimates were averaged across the 10 imputed datasets. We generated 5000 patient-level bootstrap samples from each dataset and combined the resulting estimates. Nominal 95% intervals were obtained from the 2.5th and 97.5th percentiles of this combined distribution (MI-Boot pooled percentile) [25,26].

The hypothesized relationships among FBG, baseline stent count, ISR, and clinical, anatomical, and procedural factors are shown in Figure S3 [24]. Causal interpretation requires correct temporal ordering, no unmeasured confounding of the FBG–stent count, FBG–ISR, and stent count–ISR relationships, and no stent count–ISR confounder affected by FBG [22,23,24]. Although available clinical covariates and diseased segment count were adjusted for, residual confounding by incompletely measured anatomical and treatment factors may remain. Accordingly, the estimates were interpreted as an exploratory statistical decomposition of the FBG–ISR association.

Sex-specific decomposition estimates were obtained from pooled interaction models fitted to the pre-PCI sampling subset and were considered exploratory. In addition to the relevant main effects, the mediator model included FBG × sex, and the outcome model included FBG × sex and total stent count × sex. Sex-specific ACMEs, ADEs, and TEs and women-minus-men differences were calculated within each bootstrap resample.

We repeated the decomposition after adding BMI to both models. Sensitivity analyses of Model 3 replaced FBG with HbA1c, included both FBG and HbA1c, or added diabetes status or BMI. Model 3 was also repeated in the 468 patients sampled before PCI. Multicollinearity was assessed using variance inflation factors (VIFs). E-values were calculated for the Model 3 OR and the confidence limit nearest 1 to assess sensitivity to unmeasured confounding [27].

The six imaging findings were compared between patients with and without ISR using Fisher’s exact tests. Benjamini–Hochberg correction was applied separately to the OCT/IVUS and OCT-available analyses.

Trends in underexpansion across stent-count categories (1, 2, 3, and ≥4) were assessed using the Cochran–Armitage test. Univariable logistic regression was used to estimate the association per additional stent.

The association between stent underexpansion and ISR was assessed using logistic regression. In these imaging analyses, Model 1 was unadjusted. Model 2 adjusted for total stent count. Model 3 adjusted for total stent count, age, sex, FBG, and follow-up duration. For sensitivity analyses, Model 3 was refitted after excluding patients with prior stent implantation or restricting the sample to patients with OCT records. We also estimated sex-specific associations and tested interactions with sex.

Regression and interaction tests were two-sided, with *p* < 0.05 considered statistically significant. Analyses were performed using R version 4.6.1 and Python version 3.12.14. The study was reported in accordance with STROBE [28].

## 3. Results

### 3.1. Study Population and Baseline Characteristics

The final cohort included 510 patients (349 men and 161 women) with a median age of 62 [55–68] years; 217 (42.5%) had diabetes by the study definition. ISR was observed in 56 patients (11.0%). Median FBG was 5.79 [5.07–7.59] mmol/L, total stent count was 2 [1–3], diseased segment count was 3 [2–5], and follow-up duration was 12.4 [12.0–16.2] months (Table 1). FBG was sampled before baseline PCI in 468 patients, and OCT and/or IVUS records were available for 284 patients.

Compared with patients without ISR, those with ISR had higher FBG (6.78 vs. 5.71 mmol/L; *p* = 0.012), a higher prevalence of current smoking (26.8% vs. 14.8%; *p* = 0.021), more implanted stents (median 3 vs. 2; *p* < 0.001), higher B-type natriuretic peptide levels, and lower LVEF (Table 1). Diseased segment count, HbA1c, diabetes status, lesion location, and sex distribution did not differ significantly between groups. Additional laboratory parameters and discharge medications are presented in Table S7. In comparisons stratified by sex, ISR occurred in 18.0% of men with FBG ≥7.0 mmol/L and 8.0% with FBG <7.0 mmol/L; the corresponding proportions in women were 9.4% and 11.1%, respectively. Women with FBG ≥7.0 mmol/L had higher total stent counts and diseased segment counts than women with FBG <7.0 mmol/L (*p* = 0.003 and *p* = 0.020, respectively); the corresponding comparisons in men were not statistically significant (*p* = 0.598 and *p* = 0.129, respectively; Table S1).

### 3.2. Association Between FBG and ISR

In univariable logistic regression, each 1 mmol/L higher FBG was associated with ISR (odds ratio [OR] 1.123, 95% CI 1.029–1.224; *p* = 0.009; Table S2). After adjustment for age, sex, current smoking, follow-up duration, LVEF, and STEMI (Model 3), the OR was 1.100 (95% CI 1.001–1.208; *p* = 0.048; Table 2). The point estimates were similar to Firth regression (OR 1.099, 95% CI 0.9995–1.203; *p* = 0.051) and to additional adjustment for diabetes status (OR 1.099, 95% CI 0.979–1.234; *p* = 0.109), although both confidence intervals included 1 (Table 2 and Table S4A). The overall and nonlinear spline tests yielded *p* values of 0.113 and 0.405, respectively (Figure S1). Interactions with age, sex, and diabetes status were not statistically significant (*p* for interaction = 0.109, 0.164, and 0.876, respectively; Figure S2).

### 3.3. Exploratory Statistical Decomposition and Sex-Related Estimates

**Figure 3:**
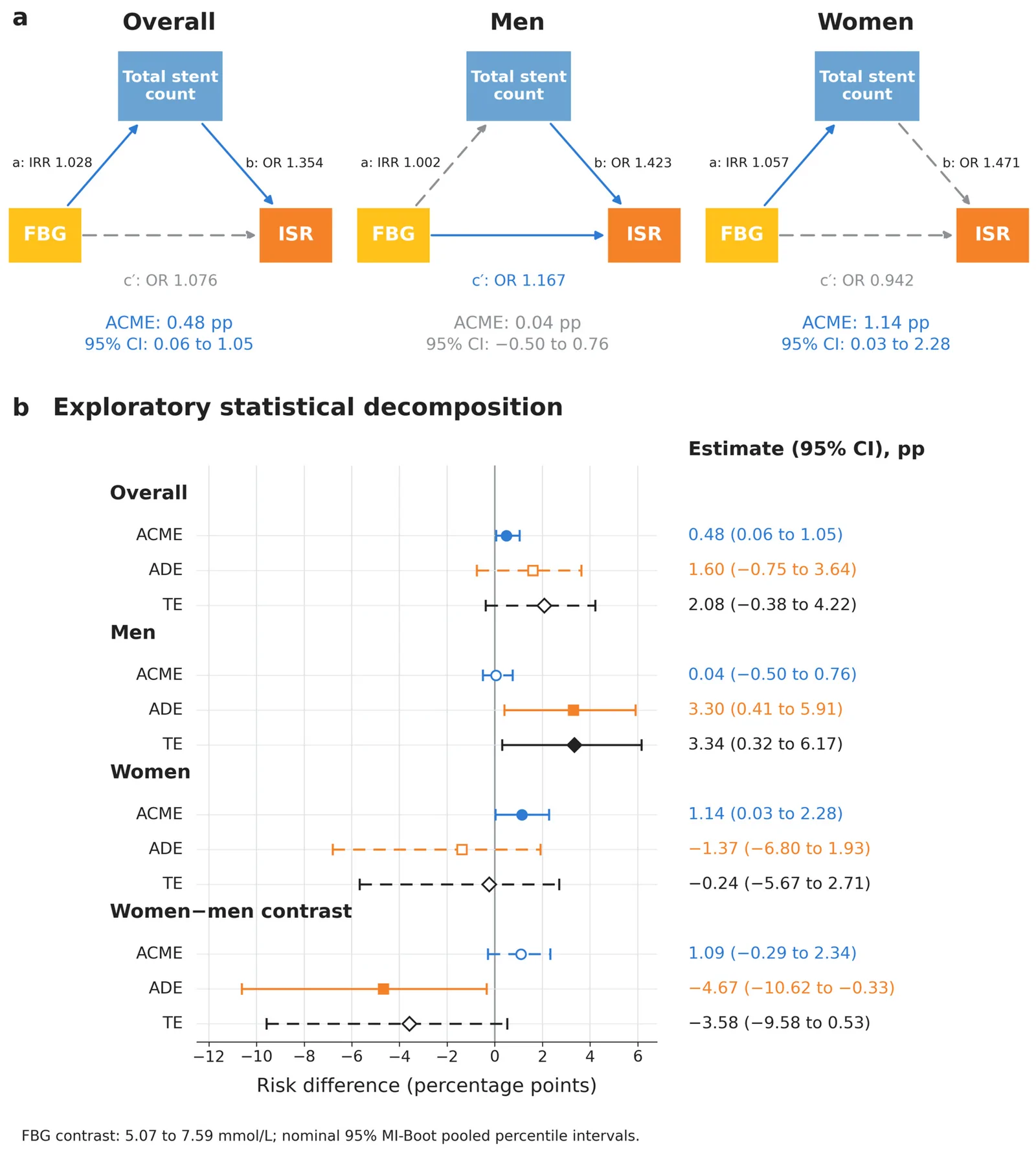
Exploratory statistical decomposition involving total stent count and sex-related estimates. (**a**) Adjusted component associations in 468 patients sampled before PCI: path a, Poisson incidence rate ratio (IRR); paths b and c′, logistic odds ratios (ORs). Path c′ is the conditional FBG–ISR association, not the ADE. (**b**) Model-based risk differences in percentage points for FBG increasing from 5.07 to 7.59 mmol/L, with nominal 95% MI-Boot pooled percentile CIs (10 imputed datasets; 5000 patient-level bootstrap resamples per dataset). Overall models omit sex interactions; sex-specific models include them. Both models were adjusted for age, sex, smoking, LVEF, STEMI, and diseased segment count; the outcome models were also adjusted for follow-up duration. In panel (**a**), solid blue arrows indicate associations with 95% CIs excluding the null value of 1, whereas dashed gray arrows indicate associations with CIs including 1. In panel (**b**), blue, orange, and black represent ACME, ADE, and TE, respectively; solid lines with filled markers indicate 95% CIs excluding zero, whereas dashed lines with open markers indicate CIs including zero.

The pre-PCI sampling subset included 468 patients with 54 ISR events. FBG was associated with total stent count (incidence rate ratio [IRR] 1.028, 95% CI 1.008–1.048; *p* = 0.007), and total stent count was associated with ISR after adjustment for FBG and the other covariates, including diseased segment count (OR 1.354, 95% CI 1.081–1.695; *p* = 0.008; Table S3). For an FBG increase from 5.07 to 7.59 mmol/L, the model-based indirect effect associated with stent count (ACME) was 0.48 percentage points (95% CI 0.06 to 1.05). The direct effect (ADE) was 1.60 percentage points (95% CI −0.75 to 3.64), and the total effect (TE) was 2.08 percentage points (95% CI −0.38 to 4.22; Figure 3 and Table S4B).

Sex-specific analyses were exploratory. The *p* values for interaction with sex were 0.003 for the FBG–stent count association, 0.073 for FBG–ISR after adjustment for stent count, and 0.890 for stent count–ISR after adjustment for FBG (Table S3). In men, the ACME, ADE, and TE were 0.04 (95% CI −0.50 to 0.76), 3.30 (95% CI 0.41 to 5.91), and 3.34 (95% CI 0.32 to 6.17) percentage points, respectively. In women, the corresponding estimates were 1.14 (95% CI 0.03 to 2.28), −1.37 (95% CI −6.80 to 1.93), and −0.24 (95% CI −5.67 to 2.71) percentage points. The positive total estimate in men was largely represented by the direct component, whereas women showed a positive indirect estimate and a negative direct estimate (Figure 3 and Table S4B). The women-minus-men contrasts were 1.09 percentage points (95% CI −0.29 to 2.34) for the indirect effect, −4.67 (95% CI −10.62 to −0.33) for the direct effect, and −3.58 (95% CI −9.58 to 0.53) for the total effect. Only the direct-effect contrast excluded zero.

### 3.4. Sensitivity Analyses

In sensitivity analyses of the overall FBG–ISR association, Firth regression yielded an FBG estimate similar to that from the conventional Model 3 (OR 1.099, 95% CI 0.9995–1.203; *p* = 0.051). When HbA1c was substituted for FBG, HbA1c was not significantly associated with ISR (OR 1.082, 95% CI 0.892–1.312; *p* = 0.425). When both glycemic measures were included, FBG remained associated with ISR (OR 1.161, 95% CI 1.013–1.332; *p* = 0.032), whereas HbA1c was not (OR 0.864, 95% CI 0.647–1.155; *p* = 0.324). Additional adjustment for diabetes status yielded an FBG estimate of similar magnitude to that in Model 3, although the confidence interval was wider and included the null (OR 1.099, 95% CI 0.979–1.234; *p* = 0.109). Additional adjustment for BMI yielded a similar FBG estimate (OR 1.105, 95% CI 1.005–1.215; *p* = 0.040; Table S4A). Restriction of Model 3 to the 468 patients sampled before PCI (54 ISR events) yielded a positive FBG estimate (OR 1.114, 95% CI 1.011–1.228; *p* = 0.029; Table S4A). No substantial multicollinearity was detected, with a maximum variance inflation factor of 2.20 (Table S4A). The E-values for the point estimate and the confidence limit closest to the null for the overall FBG–ISR association were 1.274 and 1.018, respectively. These small E-values indicate limited robustness of the overall FBG–ISR association to unmeasured confounding.

Adding BMI to both decomposition models in the pre-PCI sampling subset yielded a similar indirect-effect estimate for stent count (ACME 0.50 percentage points, 95% CI 0.07 to 1.08; Table S4B).

### 3.5. Exploratory Intravascular Imaging Findings

Among the 284 patients with evaluable imaging records, 47 (16.5%) had stent underexpansion. Its prevalence was 8.0%, 17.6%, 25.5%, and 23.9% in patients with 1, 2, 3, and ≥4 stents, respectively, with an overall increasing trend (*p* for trend = 0.0036). Each additional stent was associated with higher odds of underexpansion (OR 1.35, 95% CI 1.10–1.67; *p* = 0.005), without a significant sex interaction (*p* for interaction = 0.756; Table S5).

Stent underexpansion was associated with ISR. ISR occurred in 25.5% (12/47) of patients with underexpansion and in 6.8% (16/237) of patients without underexpansion (unadjusted OR 4.74, 95% CI 2.07–10.85; *p* < 0.001). The association remained significant after adjustment for total stent count, sex, age, FBG, and follow-up duration (OR 4.47, 95% CI 1.85–10.78; *p* = 0.001; Table S6B). Among the six imaging findings, only underexpansion was associated with ISR after Benjamini–Hochberg correction across the six Fisher’s exact tests (adjusted *p* = 0.003; Table S6A).

With the same covariate adjustment, similar associations were observed after excluding patients with prior stent implantation (OR 4.01, 95% CI 1.55–10.37; *p* = 0.004) and restricting the sample to the 225 patients with OCT records (OR 5.21, 95% CI 1.75–15.53; *p* = 0.003; Table S6C). Associations were directionally consistent in men and women, with no significant sex interaction (*p* for interaction = 0.779; Table S6C). Of the 12 patients with both stent underexpansion and ISR, 8 (66.7%) had ISR in the same coronary vessel in which stent underexpansion was detected. A formal decomposition involving the imaging findings was not performed because there were only 28 ISR events in this subset.

## 4. Discussion

In this cohort of 510 patients who underwent PCI and follow-up coronary angiography, higher FBG was modestly associated with ISR after adjustment for clinical covariates. Exploratory decomposition in the pre-PCI sampling subset identified a positive indirect effect associated with baseline stent count after adjustment for diseased segment count; additional adjustment for BMI yielded a similar estimate. The imaging analyses linked greater stent count to a higher prevalence of underexpansion and underexpansion to ISR.

Previous studies have associated admission or periprocedural hyperglycemia, higher FBG, and poor long-term glycemic control with ISR or coronary stent failure [6,7,8,9]. In our study, each 1 mmol/L higher FBG was associated with approximately 10% higher odds of ISR after adjustment for age, sex, current smoking, follow-up duration, LVEF, and STEMI. The Firth and diabetes-adjusted point estimates were similar in magnitude, although their confidence intervals included 1, indicating uncertainty about the strength of the evidence for this association. There was no clear evidence that the FBG–ISR association differed by diabetes status (*p* for interaction = 0.876). Additional adjustment for BMI produced little change in the FBG estimate, suggesting that the observed association was not materially altered by adjustment for adiposity.

Hyperglycemia may promote ISR through oxidative stress, inflammation, endothelial dysfunction, platelet activation, vascular smooth muscle cell proliferation, and extracellular matrix deposition [29,30,31,32,33,34,35]. These processes may delay vascular repair and promote neointimal formation, increasing the likelihood of recurrent narrowing within the stented segment. FBG remained associated with ISR when modeled jointly with HbA1c, whereas the HbA1c association was not statistically significant. Admission FBG may capture recent metabolic conditions, including acute stress, that are not fully represented by HbA1c [36,37].

Total stent count partly reflects the extent of coronary disease and PCI treatment. Stent count has previously been associated with ISR [10], while overlapping segments and lesion characteristics may also contribute to restenosis [2,11,12,13,14,15]. In this study, higher FBG was associated with more implanted stents, and each additional stent was associated with approximately 35% higher odds of ISR after adjustment for FBG, diseased segment count, and other covariates.

The exploratory decomposition estimated a positive indirect effect associated with stent count: 0.48 percentage points (95% CI 0.06 to 1.05) for an FBG contrast of 5.07 to 7.59 mmol/L, after adjustment for diseased segment count. Adding BMI to both models changed this estimate only slightly, to 0.50 percentage points (95% CI 0.07 to 1.08). The direct- and total-effect CIs included zero.

Exploratory sex-specific analyses showed that the positive total estimate in men was largely represented by the direct component. In women, the positive indirect estimate and negative direct estimate had opposite signs, yielding a total estimate close to zero (Figure 3 and Table S4B). The FBG–stent count association was stronger in women than in men, whereas the stent count–ISR association showed no clear sex interaction. The direct-effect estimate was larger in men; the women-minus-men ADE contrast was −4.67 percentage points (95% CI −10.62 to −0.33). However, the between-sex indirect-effect contrast included zero (1.09 percentage points, 95% CI −0.29 to 2.34). Differences in coronary caliber, lesion distribution, and vascular remodeling provide clinical context for further examination of these patterns [17,18,19,20,21]. Their reproducibility and clinical relevance warrant assessment in larger cohorts.

In the imaging subset, higher stent count was associated with a higher prevalence of underexpansion, and underexpansion was associated with ISR after adjustment for stent count and clinical covariates. The underexpansion–ISR association was similar in sensitivity analyses that used the same covariates, either excluding patients with prior stent implantation or restricting the sample to patients with OCT records. Underexpansion was also the only imaging finding associated with ISR after correction for multiple comparisons.

Stent underexpansion reduces the minimum in-stent lumen area and is an important mechanical substrate for ISR [2,11,12,13,14,15]. No significant sex interaction was detected in the imaging associations.

These findings support considering admission glycemia alongside coronary anatomy and procedural characteristics when studying ISR. FBG is readily available and reflects metabolic conditions and acute stress at admission [36]. Stent count and expansion quality provide complementary information about the extent and results of PCI treatment. Careful lesion preparation, appropriate stent sizing, optimization of expansion, and OCT or IVUS assessment when indicated are supported by guidelines and imaging-guided PCI studies [15,38,39,40,41,42].

This study has several limitations. First, the single-center retrospective design and inclusion of only patients with follow-up coronary angiography may introduce selection bias [43]. Angiography could be symptom- or ischemia-driven or scheduled, but these indications were not systematically distinguished. The observed ISR proportion therefore applies to this selected follow-up cohort. Selection related to OCT/IVUS availability may also affect the imaging findings.

Second, the decomposition analyses included 54 ISR events, of which 17 were in women, limiting the precision of sex-specific estimates and comparisons.

Third, admission FBG may reflect both prior glycemic background and acute stress, while stent count reflects disease extent and treatment decisions. We adjusted for diseased segment count, but total stent length, diameter, overlap, and detailed lesion and procedural characteristics were not systematically extracted. More stents also provide more opportunities for patient-level ISR or underexpansion to be detected, potentially contributing to the observed associations and indirect estimate. Residual and unmeasured confounding therefore remain possible.

Fourth, diabetes subtype could not be reliably classified, and glucose-lowering therapy, including SGLT2 inhibitors and GLP-1 receptor agonists, was not systematically recorded, limiting subtype- and treatment-specific assessment.

## 5. Conclusions

In this cohort of patients undergoing PCI and follow-up angiography, higher FBG was modestly associated with ISR, although statistical uncertainty varied across sensitivity analyses. In the pre-PCI sampling subset, higher FBG was associated with a higher baseline stent count, and exploratory decomposition identified a positive indirect effect associated with stent count after adjustment for measured disease extent. Additional BMI adjustment yielded a similar estimate. Exploratory sex-specific estimates showed that the positive total association in men was largely represented by the direct effect, whereas women showed a positive indirect estimate; the between-sex indirect-effect contrast remained uncertain. In the imaging subset, stent underexpansion was associated with ISR after adjustment for stent count and other covariates. These findings highlight the relevance of FBG and stent-related characteristics to observed ISR.

## Figures and Tables

**Table 1:** Baseline characteristics according to in-stent restenosis status.

Variable	No ISR (*n* = 454)	ISR (*n* = 56)	*p* Value
Demographic and clinical characteristics			
Age, years	62.5 [55.0–68.0]	60.5 [54.0–67.2]	0.332
Body mass index, kg/m^2^	25.2 [23.4–27.5]	24.6 [23.2–26.6]	0.308
Male sex, n (%)	310 (68.3)	39 (69.6)	0.836
Hypertension, n (%)	257 (56.6)	35 (62.5)	0.400
Diabetes mellitus, n (%)	189 (41.6)	28 (50.0)	0.232
Current smoking, n (%)	67 (14.8)	15 (26.8)	0.021
Prior stent implantation, n (%)	26 (5.7)	4 (7.1)	0.559
Laboratory and echocardiographic parameters			
Fasting blood glucose, mmol/L	5.71 [5.06–7.49]	6.78 [5.27–8.66]	0.012
HbA1c, %	6.00 [5.60–7.00]	6.40 [5.68–7.40]	0.141
Creatinine, μmol/L	77.0 [68.0–90.0]	78.0 [69.0–94.5]	0.349
BNP, pg/mL	150.0 [46.2–512.5]	260.0 [89.5–996.5]	0.013
LVEF, %	62.7 [61.0–64.0]	62.0 [52.8–63.0]	0.002
Systolic blood pressure, mmHg	133.0 [123.0–145.0]	136.0 [123.0–148.0]	0.607
Diastolic blood pressure, mmHg	83.0 [75.0–90.0]	80.0 [75.0–90.0]	0.505
Angiographic and procedural characteristics			
RCA disease, n (%)	354 (78.0)	44 (78.6)	0.919
LM disease, n (%)	54 (11.9)	9 (16.1)	0.370
LAD disease, n (%)	413 (91.0)	54 (96.4)	0.208
LCX disease, n (%)	307 (67.6)	42 (75.0)	0.262
Multivessel disease, n (%)	374 (82.4)	52 (92.9)	0.056
Number of diseased segments	3 [2–5]	3 [3–5]	0.229
Total stent count	2 [1–3]	3 [2–3]	<0.001
STEMI, n (%)	43 (9.5)	10 (17.9)	0.052
Follow-up duration, months	12.4 [12.0–15.2]	12.8 [11.9–27.7]	0.213

Values are median [Q1–Q3] or n (%). Continuous variables were compared by the Mann–Whitney U test; categorical variables by the chi-square test or Fisher’s exact test, as appropriate. BNP, B-type natriuretic peptide; HbA1c, glycated hemoglobin; ISR, in-stent restenosis; LAD, left anterior descending artery; LCX, left circumflex artery; LM, left main artery; LVEF, left ventricular ejection fraction; RCA, right coronary artery; STEMI, ST-segment elevation myocardial infarction. BMI summaries are based on the 508 observed values; BMI-adjusted regression analyses retained all 510 patients after multiple imputation.

**Table 2:** Association between fasting blood glucose and in-stent restenosis.

Model	OR (95% CI)	*p* Value
Model 1	1.123 (1.029–1.224)	0.009
Model 2	1.108 (1.013–1.213)	0.026
Model 3	1.100 (1.001–1.208)	0.048
Model 3 (Firth regression)	1.099 (0.9995–1.203)	0.051

Values are odds ratios (ORs) and 95% confidence intervals (CIs) per 1 mmol/L increase in fasting blood glucose (FBG). Model 1 was unadjusted. Model 2 adjusted for age, sex, current smoking, and follow-up duration. Model 3 additionally adjusted for left ventricular ejection fraction (LVEF) and ST-segment elevation myocardial infarction (STEMI). Firth regression used the Model 3 covariates. All models included 510 patients with 56 ISR events. *p* values are two-sided. Additional sensitivity analyses are reported in Table S4A. ISR, in-stent restenosis.

## Data Availability

De-identified data are available from the corresponding author on reasonable request, subject to applicable ethics and data protection requirements.

## Supplementary Materials

The following supporting information can be downloaded at: https://www.mdpi.com/article/10.3390/jcm15218685/s1, Figure S1: Fasting blood glucose and in-stent restenosis: restricted cubic spline analysis; Figure S2: Subgroup associations between fasting blood glucose and in-stent restenosis; Figure S3: Hypothesized relationships among admission FBG, baseline stent count, and ISR; Table S1: Restenosis, stent count, and diseased segments by sex and fasting blood glucose category; Table S2: Univariable associations with in-stent restenosis in the overall cohort and by sex; Table S3: Component associations and sex interactions in the exploratory decomposition; Table S4A: Sensitivity analyses of the FBG–ISR association; Table S4B: Exploratory decomposition, BMI sensitivity analysis, and sex comparisons; Table S5: Stent underexpansion according to total stent count and sex; Table S6A: Unadjusted associations of six intravascular imaging findings with in-stent restenosis; Table S6B: Adjusted associations of stent underexpansion with in-stent restenosis; Table S6C: Sensitivity and sex-specific analyses of stent underexpansion and in-stent restenosis; Table S7: Additional baseline laboratory parameters and discharge medications according to in-stent restenosis status.
