# Supplementary material for: Glycemic Status, Stent Count, and In-Stent Restenosis: Exploratory Decomposition and Sex-Related Analyses in a Retrospective Cohort
Source: J Clin Med. 2026 Nov 7;15(21):8685. doi: 10.3390/jcm15218685 (PMC13764044; doi:10.3390/jcm15218685)
Supplement: Supplementary file 1 [file jcm-15-08685-s001.zip › jcm-4603315-supplementary.pdf]

Supplementary Information

Supplementary tables and figures

Glycemic Status, Stent Count, and In-Stent Restenosis: Exploratory Decomposition and Sex-Related Analyses in a Retrospective Cohort

Yuan Wang[1], Yan Zhuang[1], Liao Zhang[1], Haiyan Song[1]

[1] Department of Endocrinology and Metabolism, The Second Affiliated Hospital of Harbin Medical University, Harbin 150001, Heilongjiang, China

Contents

Supplementary Table S1. Restenosis, stent count, and diseased segments by sex and fasting blood glucose category

Supplementary Table S2. Univariable associations with in-stent restenosis in the overall cohort and by sex

Supplementary Table S3. Component associations and sex interactions in the exploratory decomposition

Supplementary Table S4A. Sensitivity analyses of the FBG–ISR association

Supplementary Table S4B. Exploratory decomposition, BMI sensitivity analysis, and sex comparisons

Supplementary Table S5. Stent underexpansion according to total stent count and sex

Supplementary Table S6A. Unadjusted associations of six intravascular imaging findings with in-stent restenosis

Supplementary Table S6B. Adjusted associations of stent underexpansion with in-stent restenosis

Supplementary Table S6C. Sensitivity and sex-specific analyses of stent underexpansion and in-stent restenosis

Supplementary Table S7. Additional baseline laboratory parameters and discharge medications according to in-stent restenosis status

Supplementary Fig. S1. Fasting blood glucose and in-stent restenosis: restricted cubic spline analysis

Supplementary Fig. S2. Subgroup associations between fasting blood glucose and in-stent restenosis

Supplementary Fig. S3. Hypothesized relationships among admission FBG, baseline stent count, and ISR.

Supplementary Table S1. Restenosis, stent count, and diseased segments by sex and fasting blood glucose category

Characteristic	Overall (n = 510)	Men			Women		
		FBG <7.0 mmol/L (n = 238)	FBG ≥7.0 mmol/L (n = 111)	P value	FBG <7.0 mmol/L (n = 108)	FBG ≥7.0 mmol/L (n = 53)	P value
ISR, n (%)	56 (11.0)	19 (8.0)	20 (18.0)	0.010	12 (11.1)	5 (9.4)	1.000
Total stent count, median [IQR]	2 [1–3]	2 [1–3]	2 [1–3]	0.598	2 [1–2]	2 [1–4]	0.003
Number of diseased segments, median [IQR]	3 [2–5]	3 [2–5]	4 [2–5]	0.129	3 [2–4]	4 [2–5]	0.020

Values are n (%) or median [IQR]. Comparisons are between the two fasting blood glucose categories within each sex. ISR was compared using the Fisher exact test; total stent count and diseased segment count were compared using the Mann–Whitney U test. FBG, fasting blood glucose; IQR, interquartile range; ISR, in-stent restenosis.

Supplementary Table S2. Univariable associations with in-stent restenosis in the overall cohort and by sex

	Overall		Men		Women	
**Variable**	**OR (95% CI)**	**P value**	**OR (95% CI)**	**P value**	**OR (95% CI)**	**P value**
FBG, per 1 mmol/L	1.123 (1.029–1.224)	0.009	1.178 (1.049–1.322)	0.006	1.064 (0.924–1.225)	0.388
HbA1c, per 1%	1.123 (0.933–1.352)	0.221	1.092 (0.858–1.390)	0.474	1.193 (0.883–1.611)	0.250
Diabetes mellitus	1.402 (0.804–2.445)	0.234	1.546 (0.792–3.016)	0.202	1.157 (0.423–3.166)	0.777
Stent count, per stent	1.375 (1.150–1.644)	<0.001	1.334 (1.071–1.661)	0.010	1.462 (1.074–1.990)	0.016
Number of diseased segments, per segment	1.112 (0.967–1.280)	0.137	1.081 (0.914–1.279)	0.362	1.186 (0.922–1.525)	0.185
Current smoking	2.113 (1.108–4.031)	0.023	2.382 (1.168–4.861)	0.017	1.062 (0.125–9.052)	0.956
LVEF, per 1%	0.940 (0.912–0.968)	<0.001	0.944 (0.912–0.977)	0.001	0.926 (0.870–0.984)	0.014
ST-segment elevation myocardial infarction	2.078 (0.979–4.410)	0.057	1.900 (0.776–4.655)	0.160	2.591 (0.645–10.405)	0.180
Follow-up duration, per month	1.027 (1.009–1.046)	0.003	1.046 (1.026–1.067)	<0.001	1.032 (1.001–1.064)	0.041
Age, per year	0.989 (0.960–1.018)	0.440	0.985 (0.951–1.020)	0.384	1.000 (0.942–1.063)	0.987
Female sex (vs male)	0.938 (0.513–1.715)	0.836	—	—	—	—

Odds ratios (ORs) and 95% confidence intervals (CIs) are from univariable logistic regression models. Sex-specific models were fitted separately in men and women, with sex omitted as a predictor. P values are from two-sided Wald tests. FBG, fasting blood glucose; HbA1c, glycated hemoglobin; ISR, in-stent restenosis; LVEF, left ventricular ejection fraction.

Supplementary Table S3. Component associations and sex interactions in the exploratory decomposition

Pathway	Effect metric	Overall		Male		Female		P interaction
		Estimate (95% CI)	P value	Estimate (95% CI)	P value	Estimate (95% CI)	P value	
FBG → Stent count (a)	IRR	1.028 (1.008–1.048)	0.007	1.002 (0.977–1.028)	0.878	1.057 (1.030–1.085)	<0.001	0.003
Stent count → ISR (adjusted for FBG; b)	OR	1.354 (1.081–1.695)	0.008	1.423 (1.087–1.864)	0.010	1.471 (0.977–2.215)	0.065	0.890
FBG → ISR (without stent count; c)	OR	1.098 (0.993–1.215)	0.067	1.158 (1.018–1.318)	0.026	1.022 (0.873–1.195)	0.790	0.222
FBG → ISR (adjusted for stent count; c′)	OR	1.076 (0.970–1.193)	0.165	1.167 (1.025–1.329)	0.020	0.942 (0.771–1.149)	0.554	0.073

Analyses included 468 patients (54 ISR events). Models adjusted for age, sex, current smoking, LVEF, STEMI, and diseased segment count; ISR models also adjusted for follow-up duration. Overall models had no sex interactions. Sex-specific models included FBG × sex; models containing stent count also included stent count × sex. Estimates were pooled across 10 imputations using Rubin's rules. P for interaction compares women and men. FBG and stent count estimates are per 1 mmol/L and per stent, respectively. FBG, fasting blood glucose; IRR, incidence rate ratio; ISR, in-stent restenosis; LVEF, left ventricular ejection fraction; OR, odds ratio; STEMI, ST-segment elevation myocardial infarction.

Supplementary Table S4A. Sensitivity analyses of the FBG–ISR association

Model	Variable	OR (95% CI)	P value	Max VIF
Model 3	FBG	1.100 (1.001–1.208)	0.048	1.14
Model 3 + diabetes	FBG	1.099 (0.979–1.234)	0.109	1.61
	Diabetes mellitus	1.029 (0.496–2.137)	0.938	
Model 3 + HbA1c	FBG	1.161 (1.013–1.332)	0.032	2.20
	HbA1c	0.864 (0.647–1.155)	0.324	
HbA1c replacing FBG	HbA1c	1.082 (0.892–1.312)	0.425	1.10
Model 3 + BMI (n = 510)	FBG	1.105 (1.005–1.215)	0.040	1.19
	BMI	0.941 (0.852–1.038)	0.223	
Model 3: FBG sampled before PCI (n = 468; ISR = 54)	FBG	1.114 (1.011–1.228)	0.029	1.18

Model 3 adjusted for age, sex, current smoking, follow-up duration, LVEF, and STEMI. Sensitivity models modified Model 3 as indicated. Analyses used 510 patients (56 ISR events), except the pre-PCI analysis (468 patients; 54 events). Estimates were pooled across 10 imputations using Rubin's rules. FBG, HbA1c, and BMI estimates are per 1 mmol/L, 1 percentage point, and 1 kg/m², respectively; absence of diabetes is the reference category. Max VIF is the largest VIF among model terms; for BMI and pre-PCI analyses, the largest value across imputations is shown. BMI, body mass index; FBG, fasting blood glucose; HbA1c, glycated hemoglobin; ISR, in-stent restenosis; LVEF, left ventricular ejection fraction; OR, odds ratio; STEMI, ST-segment elevation myocardial infarction; VIF, variance inflation factor.

Supplementary Table S4B. Exploratory decomposition, BMI sensitivity analysis, and sex comparisons

Analysis	Patients (ISR events)	Risk difference, percentage points (nominal 95% CI)		
		ACME (indirect)	ADE (direct)	TE (total)
Overall	468 (54)	0.48 (0.06 to 1.05)	1.60 (−0.75 to 3.64)	2.08 (−0.38 to 4.22)
Overall, BMI added (sensitivity)	468 (54)	0.50 (0.07 to 1.08)	1.66 (−0.71 to 3.75)	2.16 (−0.32 to 4.36)
Men	321 (37)	0.04 (−0.50 to 0.76)	3.30 (0.41 to 5.91)	3.34 (0.32 to 6.17)
Women	147 (17)	1.14 (0.03 to 2.28)	−1.37 (−6.80 to 1.93)	−0.24 (−5.67 to 2.71)
Women minus men		1.09 (−0.29 to 2.34)	−4.67 (−10.62 to −0.33)	−3.58 (−9.58 to 0.53)

Effects are for FBG increasing from 5.07 to 7.59 mmol/L, estimated by g-computation; TE = ACME + ADE (up to rounding). Both models adjusted for age, sex, current smoking, LVEF, STEMI, and diseased segment count; the outcome model also adjusted for follow-up duration. BMI was added to both models in the sensitivity row. Sex-specific estimates used the interaction models in Table S3; the difference is women minus men. Missing LVEF and BMI were multiply imputed; CIs are MI-Boot pooled percentile intervals (see Methods). ACME, ADE, and TE are conventional labels for model-based indirect, direct, and total effects; interpretation is exploratory and associational. BMI, body mass index; CI, confidence interval; FBG, fasting blood glucose; ISR, in-stent restenosis; LVEF, left ventricular ejection fraction; STEMI, ST-segment elevation myocardial infarction.

Supplementary Table S5. Stent underexpansion according to total stent count and sex

Stent count	Overall (n = 284)	Male (n = 203)	Female (n = 81)
1 stent	8/100 (8.0%)	5/79 (6.3%)	3/21 (14.3%)
2 stents	16/91 (17.6%)	10/56 (17.9%)	6/35 (17.1%)
3 stents	12/47 (25.5%)	9/34 (26.5%)	3/13 (23.1%)
≥4 stents	11/46 (23.9%)	6/34 (17.6%)	5/12 (41.7%)
P for trend	0.0036	0.024	0.066
Per additional stent, OR (95% CI)	1.35 (1.10–1.67)	1.31 (1.01–1.71)	1.41 (0.99–2.02)
Per additional stent, P value	0.005	0.043	0.060

Values are patients with underexpansion/total patients in the subgroup (%); n = 284. Trends were assessed using the Cochran–Armitage test. Odds ratios were estimated using univariable logistic regression with total stent count as a continuous variable. P for stent count × sex interaction = 0.756. CI, confidence interval; OR, odds ratio. This is an available-imaging subset, not a random sample of the full cohort; the patient-level findings are exploratory. Underexpansion denotes its presence in any assessed stent in a patient; patients with more stents have more opportunities to meet this definition.

Supplementary Table S6A. Unadjusted associations of six intravascular imaging findings with in-stent restenosis

Imaging finding	Finding present ISR, n/N (%)	Finding absent ISR, n/N (%)	OR (95% CI)	P value	BH-adjusted P value
**OCT and/or IVUS (n = 284)**					
Stent underexpansion	12/47 (25.5%)	16/237 (6.8%)	4.74 (2.07–10.85)	<0.001	0.003
Stent malapposition	2/23 (8.7%)	26/261 (10.0%)	0.86 (0.19–3.88)	1.000	1.000
Stent-edge dissection/tear	3/42 (7.1%)	25/242 (10.3%)	0.67 (0.19–2.32)	0.779	0.989
Tissue prolapse	10/146 (6.8%)	18/138 (13.0%)	0.49 (0.22–1.10)	0.110	0.330
Plaque rupture	4/31 (12.9%)	24/253 (9.5%)	1.41 (0.46–4.38)	0.525	0.989
Thrombus	8/76 (10.5%)	20/208 (9.6%)	1.11 (0.47–2.63)	0.824	0.989
**OCT available (n = 225)**					
Stent underexpansion	8/35 (22.9%)	10/190 (5.3%)	5.33 (1.93–14.70)	0.002	0.013
Stent malapposition	1/20 (5.0%)	17/205 (8.3%)	0.58 (0.07–4.62)	1.000	1.000
Stent-edge dissection/tear	3/40 (7.5%)	15/185 (8.1%)	0.92 (0.25–3.34)	1.000	1.000
Tissue prolapse	9/134 (6.7%)	9/91 (9.9%)	0.66 (0.25–1.72)	0.456	0.908
Plaque rupture	4/27 (14.8%)	14/198 (7.1%)	2.29 (0.69–7.53)	0.245	0.734
Thrombus	7/74 (9.5%)	11/151 (7.3%)	1.33 (0.49–3.58)	0.605	0.908

Finding-present and finding-absent cells show ISR events/total patients (%). Odds ratios are unadjusted. P values are from two-sided Fisher exact tests. The six P values were adjusted separately within the OCT and/or IVUS analysis set and the OCT-available analysis set using the Benjamini–Hochberg procedure. CI, confidence interval; ISR, in-stent restenosis; IVUS, intravascular ultrasound; OCT, optical coherence tomography; OR, odds ratio.

Supplementary Table S6B. Adjusted associations of stent underexpansion with in-stent restenosis

Model	Covariates adjusted	OR (95% CI)	P value
Model 1	Unadjusted	4.74 (2.07–10.85)	<0.001
Model 2	Total stent count	4.19 (1.80–9.76)	0.001
Model 3	Model 2 + sex, age, FBG, and follow-up duration	4.47 (1.85–10.78)	0.001

Odds ratios compare patients with and without stent underexpansion; n = 284, with 28 ISR events. Model 1 was unadjusted. Model 2 adjusted for total stent count. Model 3 additionally adjusted for sex, age, fasting blood glucose, and follow-up duration. CI, confidence interval; FBG, fasting blood glucose; ISR, in-stent restenosis; OR, odds ratio.

Supplementary Table S6C. Sensitivity and sex-specific analyses of stent underexpansion and in-stent restenosis

Analysis	Subgroup	n (ISR events)	OR (95% CI)	P value	P interaction
Excluding prior stent patients	—	264 (25)	4.01 (1.55–10.37)	0.004	—
OCT-available subset	—	225 (18)	5.21 (1.75–15.53)	0.003	—
Sex-stratified analysis	Male	203 (21)	4.10 (1.51–11.13)	0.006	0.779
	Female	81 (7)	5.04 (0.95–26.68)	0.057	—

Sensitivity analyses excluding patients with prior stent implantation or restricting the sample to patients with OCT records adjusted for total stent count, age, sex, fasting blood glucose, and follow-up duration. Sex-specific odds ratios are adjusted for total stent count. The interaction P value is from a model including underexpansion, sex, total stent count, and underexpansion × sex. CI, confidence interval; ISR, in-stent restenosis; OR, odds ratio. Sex-specific estimates and the interaction test were exploratory.

Supplementary Table S7. Additional baseline laboratory parameters and discharge medications according to in-stent restenosis status

Variable	No ISR (n = 454)	ISR (n = 56)	P value
**Additional laboratory parameters**			
Random blood glucose, mmol/L	6.70 [5.40–8.91]	7.42 [5.80–10.84]	0.101
Uric acid, μmol/L	322.0 [272.9–380.1]	335.6 [292.6–390.6]	0.348
Creatine kinase, U/L	95.0 [65.5–150.0]	99.0 [74.0–220.5]	0.199
CK-MB, U/L	1.5 [0.6–11.9]	1.9 [0.6–11.1]	0.886
Total cholesterol, mmol/L	4.04 [3.30–4.81]	4.15 [3.54–4.79]	0.613
Triglycerides, mmol/L	1.47 [1.02–2.00]	1.36 [1.09–1.99]	0.761
HDL cholesterol, mmol/L	1.01 [0.86–1.19]	1.02 [0.84–1.19]	0.888
LDL cholesterol, mmol/L	2.53 [1.86–3.18]	2.53 [2.20–3.20]	0.479
**Discharge medications**			
Aspirin or indobufen, n (%)	453 (99.8)	56 (100.0)	1.000
P2Y12 inhibitor, n (%)	452 (99.6)	56 (100.0)	1.000
Statin, n (%)	452 (99.6)	55 (98.2)	0.295
Isosorbide mononitrate, n (%)	210 (46.3)	29 (51.8)	0.434
ACEI/ARNI/ARB, n (%)	117 (25.8)	16 (28.6)	0.652
Beta-blocker, n (%)	148 (32.6)	20 (35.7)	0.640
Calcium channel blocker, n (%)	72 (15.9)	8 (14.3)	0.760

Values are median [Q1–Q3] or n (%). Continuous variables were compared by the Mann–Whitney U test; categorical variables by the chi-square test or Fisher exact test, as appropriate. ACEI, angiotensin-converting enzyme inhibitor; ARB, angiotensin receptor blocker; ARNI, angiotensin receptor–neprilysin inhibitor; CK-MB, creatine kinase-MB isoform; HDL-C, high-density lipoprotein cholesterol; ISR, in-stent restenosis; LDL-C, low-density lipoprotein cholesterol.

Supplementary Fig. S1. Fasting blood glucose and in-stent restenosis: restricted cubic spline analysis

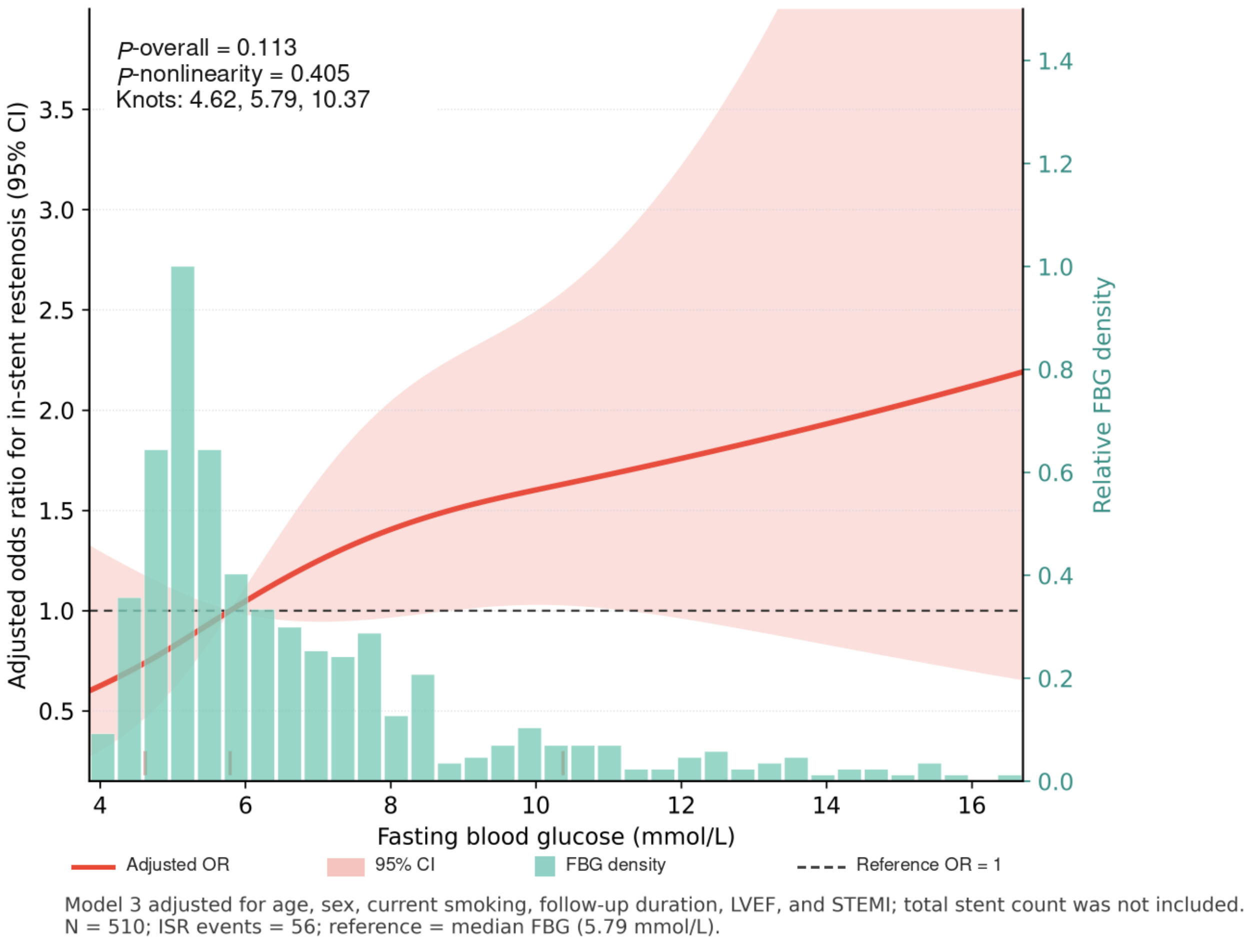

Knots were placed at 4.62, 5.79, and 10.37 mmol/L, with 5.79 mmol/L as the reference. Model 3 adjusted for age, sex, current smoking, follow-up duration, left ventricular ejection fraction, and ST-segment elevation myocardial infarction, without total stent count. The overall and nonlinear *P* values were obtained from Wald tests. FBG, fasting blood glucose; ISR, in-stent restenosis; OR, odds ratio.

Supplementary Fig. S2. Subgroup analysis of the association between fasting blood glucose and in-stent restenosis.

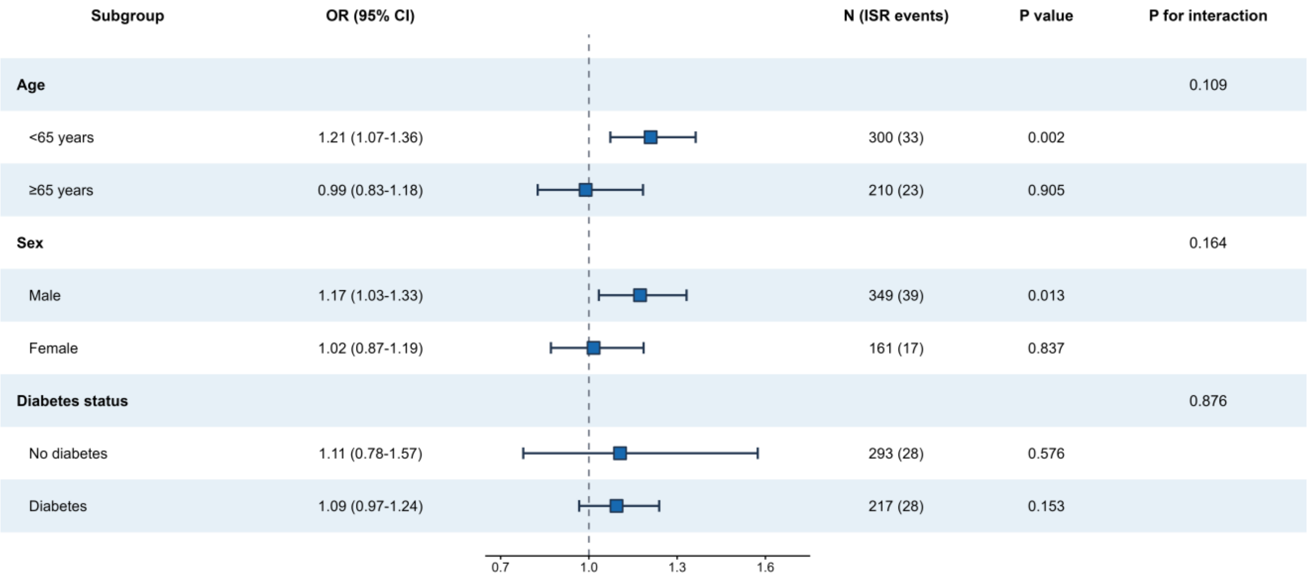

Odds ratios (ORs) and 95% confidence intervals (CIs) are presented for each 1 mmol/L increase in fasting blood glucose. The estimates were derived from multivariable logistic regression models, adjusted for age, sex, current smoking, follow-up duration, left ventricular ejection fraction, and ST-segment elevation myocardial infarction, where applicable. *P* values indicate associations within each subgroup. *P* values for interaction were obtained by adding an interaction term between fasting blood glucose and the relevant subgroup variable to Model 3. "N" denotes the number of participants, with ISR events indicated in parentheses. The dashed vertical line represents an OR of 1.0. ISR, in-stent restenosis. Subgroup estimates and interaction tests are exploratory; sex-related comparisons are based on 17 ISR events in women.

Supplementary Fig. S3. Hypothesized relationships among admission FBG, baseline stent count, and ISR.

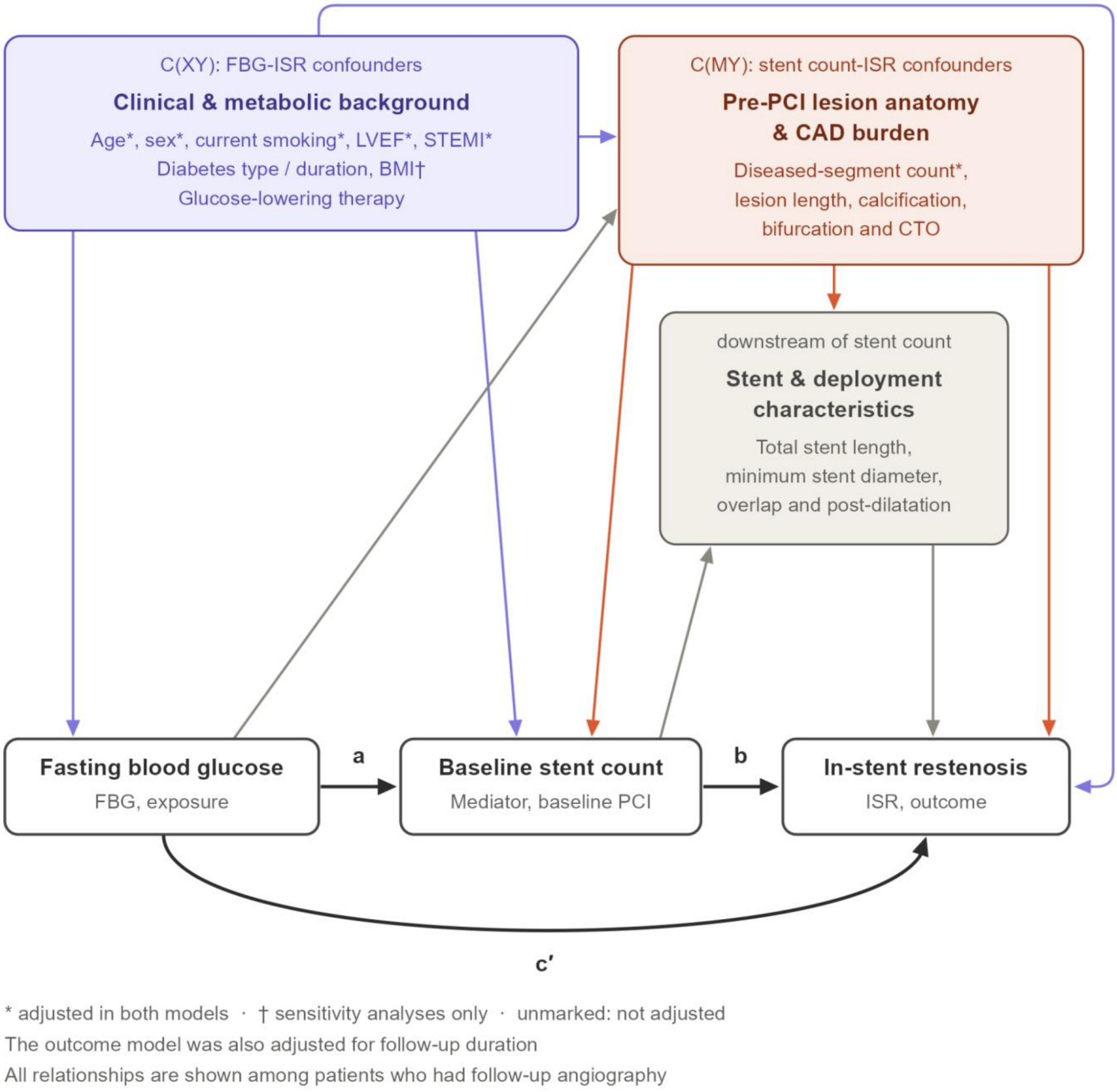

Arrows show hypotheses rather than relationships established by these data. C(XY) and C(MY) group clinical and metabolic background and pre-PCI coronary anatomy, respectively. Stent and deployment characteristics are shown downstream of stent count. *Adjusted in both models; †included only in BMI sensitivity analyses; unmarked factors were not adjusted for. The outcome model also adjusted for follow-up duration. CAD, coronary artery disease; CTO, chronic total occlusion; FBG, fasting blood glucose; ISR, in-stent restenosis; LVEF, left ventricular ejection fraction; PCI, percutaneous coronary intervention; STEMI, ST-segment elevation myocardial infarction.
